# Education-Related Parameters in High Myopia: Adults versus School Children

**DOI:** 10.1371/journal.pone.0154554

**Published:** 2016-05-06

**Authors:** Jost B. Jonas, Liang Xu, Ya Xing Wang, Hong Sheng Bi, Jian Feng Wu, Wen Jun Jiang, Vinay Nangia, Ajit Sinha, Dan Zhu, Yong Tao, Yin Guo, Qi Sheng You, Li Juan Wu, Li Xin Tao, Xiu Hua Guo, Kyoko Ohno-Matsui, Songhomitra Panda-Jonas

**Affiliations:** 1 Beijing Institute of Ophthalmology, Beijing Tongren Eye Center, Beijing Tongren Hospital, Capital Medical University, Beijing Ophthalmology and Visual Science Key Lab, Beijing, China; 2 Department of Ophthalmology, Medical Faculty Mannheim of the Ruprecht-Karls-University of Heidelberg, Heidelberg, Germany; 3 Shandong University of Traditional Chinese Medicine, Jinan, Shandong, China; 4 Eye Institute of Shandong University of Traditional Chinese Medicine, Jinan, Shandong, China; 5 Suraj Eye Institute, Nagpur, India; 6 The Affiliated Hospital of Inner Mongolia Medical University, Hohhot, Inner Mongolia, China; 7 Department of Ophthalmology, People’s Hospital, Peking University, & Key Laboratory of Vision Loss and Restoration, Ministry of Education, Beijing, China; 8 Tongren Eye Care Center, Beijing Tongren Hospital, Capital Medical University, Beijing, China; 9 School of Public Health, Capital Medical University, Beijing, China; 10 Beijing Key Laboratory of Epidemiology, Capital Medical University, Beijing, China; 11 Department of Ophthalmology and Visual Science, Tokyo Medical and Dental University, Tokyo, Japan; Medical College of Soochow University, CHINA

## Abstract

**Purpose:**

Since high myopia in the younger generation may differ etiologically from high myopia in older generations, we examined whether education-related parameters differ between high myopia in today´s school children and high pathological myopia in today´s elderly generation.

**Methods:**

The investigation included the adult populations of the population-based Beijing Eye Study (BES) (3468 adults;mean age:64.6±9.8years;range:50–93years) and Central India Eye and Medical Study (CIEMS) (4711 adults;age:49.±13.2years;range:30–100years), and the children and teenager populations of the Shandong Children Eye Study (SCES) (6026 children;age:9.7±3.3years;range:4–18years;cycloplegic refractometry), Gobi Desert Children Eye Study (1565;age:11.9±3.5years;range:6–21 years;cycloplegic refractometry), Beijing Pediatric Eye Study (681 children;age:7.7±1.6years;range:5–13 years;non-cycloplegic refractometry,calculation of axial length to corneal curvature radius ratio), Beijing Children Eye Study (15066 children;age:13.2±3.4years;range:7–18years;non-cycloplegic refractometry), Beijing High School Teenager Eye Study (4677 children;age:16.9±0.7years;range:16–18years;non-cycloplegic refractometry).

**Results:**

In the BES and CIEMS, educational level did not differ significantly between, or was significantly lower in the highly myopic group (myopic refractive error ≥6 diopters) than in the non-highly myopic group. In all non-adult study populations, higher prevalence of high myopia was significantly associated with higher degree of education related parameters such as attendance of high-level schools, and more time spent for indoors near work versus time spent outdoors.

**Conclusions:**

Comparing associations of old or genetic high myopia in adults with new or acquired high myopia in school children revealed that education-related parameters did not show a clear association with old or genetic high myopia, while in contrast, new high myopia showed strong associations with education. It confirms previous studies that the two forms of high myopia not only differed in age of onset, but also in associations with education as well. The data support the notion of two types of high myopia. Future studies may assess whether the risk of pathologic myopic maculopathy and high myopia associated open-angle glaucoma differs between both types of high myopia.

## Introduction

The marked increase in the prevalence of myopia in particularly in East and Southeast Asia has raised the fear of a profound increase in the prevalence of myopia related blindness [1–4], which can be due to the sequelae of myopic retinopathy, high myopia-related open-angle glaucoma and other features [5,6]. Several investigations have already assessed which factors were associated with the rise in the prevalence of myopia. These parameters included a higher level of education of children and parents, a change in the lifestyle including less time spent outdoors and more time spent indoors during childhood and adolescence, urban region of habitation, and higher socioeconomic background of the parents [7–10]. It has remained unclear whether the type of myopia observed in today´s highly myopic school children and which may be called “acquired high myopia” is similar to the type of myopia seen in today´s middle aged and elderly highly myopic population in which myopia is a major risk factor for visually disabling disorders and which may be called “classical genetic high myopia” [4,7].

Studies by Lin and others suggested that the prevalence of high myopia has profoundly increased in the period from 1983 onwards, and that the prevalence started to increase markedly from the children´s age of 11–13 years [10–12]. Liang et al. found that myopic children with highly myopic parents were more likely to have an early onset of myopia with an odds ratio of 2.61 [11]. A study by Xiang and colleagues on Chinese children revealed that a small portion of children were highly myopic from the age of 5 years onwards, while a marked increase in the prevalence of high myopia was observed in the group of children aged 12+ years [12]. These investigations confirmed previous observations that the new form of high myopia seen in more recent birth cohorts in East and Southeast Asia (“acquired high myopia”) showed a distinctive environmental etiology (e.g., higher level of education, more near work and less time spent outdoors) and a relatively late onset in contrast to the more hereditary form of high myopia (“classical genetic high myopia”), which started at a relatively young age, which had been predominant up to a time of about 20 years ago, and which appeared to be less dependent on environmental parameters [7]. Due to the presumably different etiology, it has been suggested that both forms of high myopia may differ in the eventual development of pathological myopia in later life [7]. This appeared to be likely, because at least some forms of genetic high myopia involved mutations directly affecting the sclera and thus making these eyes more susceptible to the development of pathologies such as myopic staphyloma and extreme axial elongation [13].

In attempt to further elucidate potential differences in the etiologic background between both types of myopia, we conducted this study to compare highly myopic young individuals and highly myopic adult individuals by assessing associations of the prevalence of high myopia with parameters of education and with ocular biometric variables.

## Methods

The Beijing Eye Study 2011 was a population-based cross-sectional study in Northern China [14]. The Medical Ethics Committee of the Beijing Tongren Hospital approved the study protocol and all participants gave informed written consent, according to the Declaration of Helsinki. Out of 4403 eligible individuals with an age of 50+ years, 3468 individuals (78.8%) participated with a mean age of 64.6 ± 9.8 years (median, 64 years; range, 50–93 years). More than 99.5% of the study population was Han Chinese. The socioeconomic status was assessed with questions about the level of education, occupation, and family income. The level of education was categorized into the stages of “illiteracy”, “half illiteracy with knowledge of some Chinese words”, “primary school education”, “middle school education”, and “college or higher education”. Cycloplegia was not applied for refractometry.

The Central India Eye and Medical Study was a population-based cross-sectional study conducted in a rural region in Central India close to the so called tribal belt [15]. The Medical Ethics Committee of the Medical Faculty Mannheim of the Ruprecht-Karls-University Heidelberg and the ethical committee of Suraj Eye Institute / Nagpur approved the study and all participants gave informed written consent, according to the Declaration of Helsinki. Inclusion criterion was an age of 30+ years. Out of eligible 5885 individuals, 4711 (80.1%) subjects were included into the study with a mean age of 49.1 ± 13.2 years (range: 30–100 years). The level of education was graded into five categories (illiterate; school visit up the 5th standard; school visit up to 6th to 8th standard; school attendance up to 9th and 12th standard; graduation or higher). The participants of both studies, the Beijing Eye Study and the Central India Eye and Medical Study, underwent a similar series of examinations including a detailed interview by trained health staff asking similar questions on family status, level of education, income, and known major systemic diseases, and non-cycloplegic refractometry.

The Shandong Children Eye Study (SCES) was a school-based cross-sectional study in East China and was performed in the city of Weihai in the eastern part of the coastal province of Shandong, and in the rural areas of Guanxian in the western region of Shandong [16]. The Ethics Board of the Eye Institute of the Shandong University of Traditional Chinese Medicine and the local Administration of the Education and School Board approved the study, and the parents or guardians of the children gave written consent, according to the Declaration of Helsinki. Kindergartens, elementary schools, junior high schools and senior high schools in Guanxian and in Weihai were randomly selected for the study. Out of the eligible 6364 children, 6026 (94.7%) children with a mean age of 9.7 ± 3.3 years (range 4 to 18 years) were included. Refractive error was measured by refractometry in cylcoplegic conditions. After an initial topical anesthesia with one drop of 0.4% oxybuprocaine (Santen Co., Shiga, Japan), cycloplegia was achieved by applying 1% cyclopentolate eye drops (Alcon, Ft. Worth, Texas, USA) three times in intervals of 5 minutes.

The Gobi Desert Children Eye Study was a cross-sectional, school-based study, which was performed in the city oasis of Ejina in the Gobi desert [17]. The Ethics Board of the Affiliated Hospital of Inner Mongolia Medical University Hohhot and the local Administration of the Education and School Board of Ejina approved the study and informed written consent was obtained from the parents or guardians of all children. The study included all three available schools in Ejina with overall 1911 eligible children out of whom 1565 (81.9%) children with a mean age of 11.9 ± 3.5 years (range: 6 to 21 years) participated. Refractometry was carried out under cycloplegic conditions. Cycloplegia was achieved by instilling one drop of 1% cyclopentolate (Alcon, Ft. Worth, USA) at least three times in intervals of about 10 minutes, before auto-refraction measurements (ARK-900, NIDEK, Tokyo, Japan) was carried out.

The Beijing Pediatric Eye Study included children attending primary schools (grade 1 and grade 4) from rural regions and urban areas of Greater Beijing [18]. The study protocol was approved by the Human Research Ethics Committee of the TongRen Hospital, Capital Medical University, Beijing, and informed written consent was obtained from at least one parent of each of the participating children. The study included 681 children with a mean age of 7.7 ± 1.6 years (range: 5–13 years). The children underwent a comprehensive eye examination including auto-refractometry (auto-refractor KR-8900, Topcon, Tokyo, Japan) and subjective refractometry as well as ocular biometry by optical low-coherence reflectometry. As described in detail by Grosvenor and Scott, the ratio of axial length to corneal curvature radius (AL/CCR ratio) was used as the best available surrogate for refractive error since cycloplegic refractometry was not performed [19]. The method developed by Grosvenor and Scott in 1994 was re-addressed in the Sydney Myopia Study and in the Singapore Cohort study Of the Risk factors for Myopia (SCORM), which confirmed that the AL/CCR ratio, with major limitations, provided the best correlation to cycloplegic refraction that could be obtained using non-invasive techniques [20,21].

In a separate part, our investigations also included the results of two studies on children or teenagers whose refractive error was not measured under cycloplegic conditions and for whom ocular biometric measurements were not available. These were the Beijing Children Eye Study and the Beijing High School Teenager Eye Study [22,23]. The Beijing Children Eye Study was a population-based study conducted in the region of Greater Beijing and it was approved by the ethics committee of the Capital Medical University, the Beijing Municipal Commission of Education and the Beijing Center for Disease Control and Prevention [22]. The parents of all children gave written consent. Out of eligible 16,771 students, 15,066 (89.8%) students participated. The mean age was 13.2 ± 3.4 years (range: 7 to 18 years). The Beijing High School Teenager Eye Study was a cross-sectional school based study in Beijing [23]. The study was approved by the ethics committee of the Capital Medical University, the Beijing Municipal Commission of Education and the Beijing Center for disease Control and Prevention. It followed the tenets of Declaration of Helsinki. Written consent was obtained from the parents of all students. Out of 4798 eligible students, the study finally included 4677 (93.4%) students (2490 (53.7%) girls) with a mean age of 16.9 ± 0.7 years (range: 16–18 years). The majority of the students were Han Chinese (89.3%).

Since the necessity of cycloplegia for reliable refractometric results has previously been shown, the findings obtained in the Beijing Children Eye Study and the Beijing High School Teenager Eye Study have to be discussed with the caveat of a potential bias by the lack of cycloplegia during refractometry [24]. There has been some disagreement over whether cycloplegia is necessary for studies on adults, but a recent review by Morgan and colleagues revealed that cycloplegia was required only up to the age of about 50 years [24].

In the studies including children and teenagers, all study participants underwent an interview with similar questions. The structure of the questionnaire was comparable to the design of questionnaires which were used in previous similar investigations and most of which were derived from, or were strongly influenced by, the questionnaire developed for the Sydney Myopia Study [20]. The questionnaire included questions on the time spent outdoors with sports, time spent outdoors with leisure, time spent indoors with writing or reading, time spent indoors with watching television, and time spent indoors with playing electronic games.

A commercially available statistical software package (SPSS for Windows, version 22.0, IBM-SPSS, Chicago, IL, USA) was used for the statistical analysis. We calculated the prevalence of high myopia (defined as a myopic refractive of 6 or more diopters). In univariate analysis we analyzed associations between the presence of high myopia and other ocular and systemic parameters. We then performed a multivariate binary regression analysis, with the presence of high myopia as dependent variable and as independent variables all those parameters which were significantly associated with the presence of high myopia in the univariate analysis. Besides parameters on the level of education, the socioeconomic background and on time spent indoors / outdoors, these multivariate analyses included other ocular and systemic variables such as age, gender, body height, region of habitation, and intraocular pressure, to mention a few. Odds ratios (OR) and 95% confidence intervals (95%CI) were calculated. All *P*-values were 2-sided and were considered statistically significant when the values were less than 0.05.

## Results

**Fig 1:**
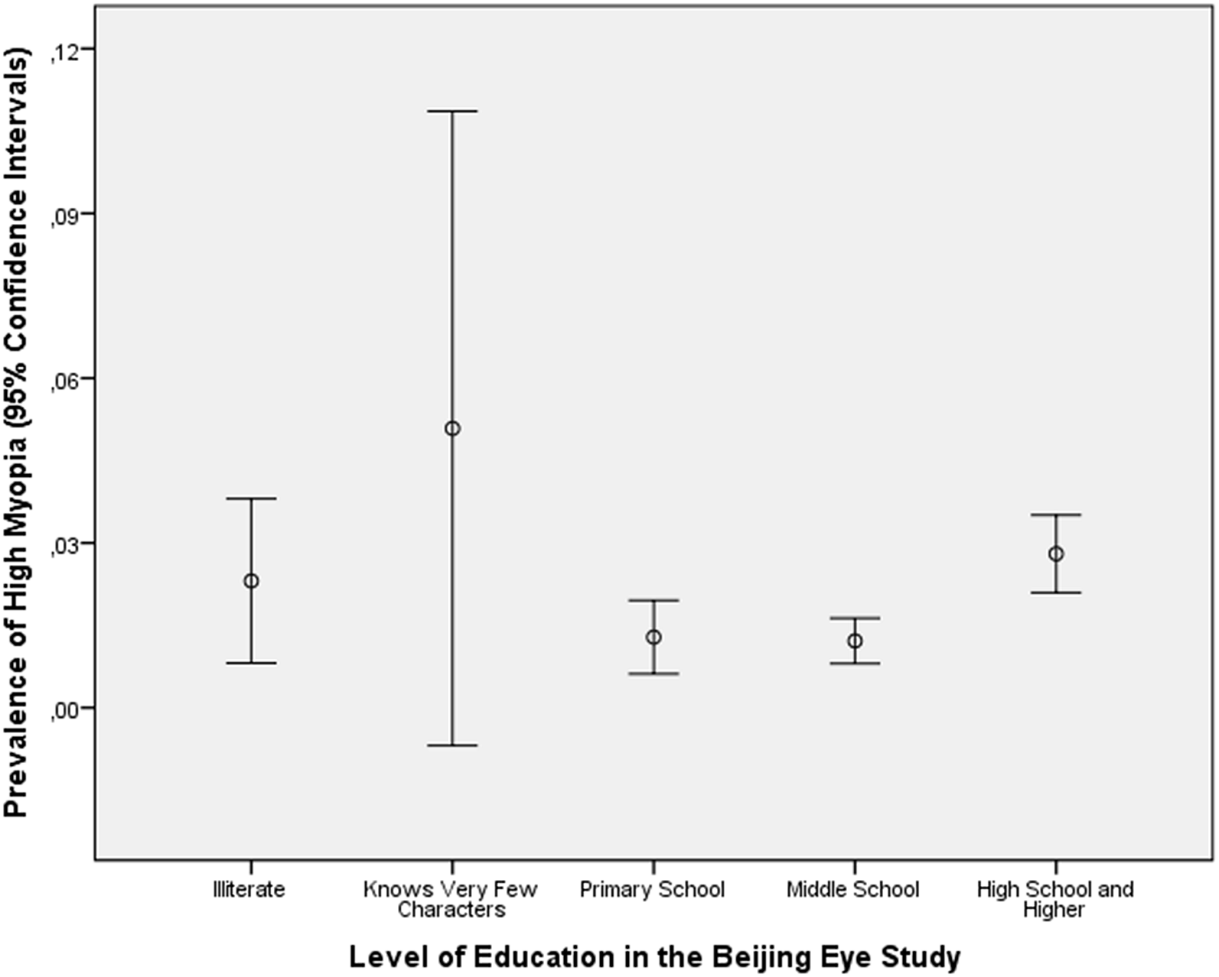
Distribution of the Prevalence of High Myopia (Defined as a Myopic Refractive Error of 6 or more Diopters), Stratified by the Level of Education in the Beijing Eye Study.

In the Beijing Eye Study, the level of education did not differ significantly between the highly myopic group and the non-highly myopic group (4.0 ± 1.3 versus 4.0 ± 1.1; *P* = 0.99) (Fig 1). Correspondingly, the prevalence of high myopia was not significantly (*P* = 0.13; standardized regression coefficient beta: 0.02) associated with the level of education.

**Fig 2:**
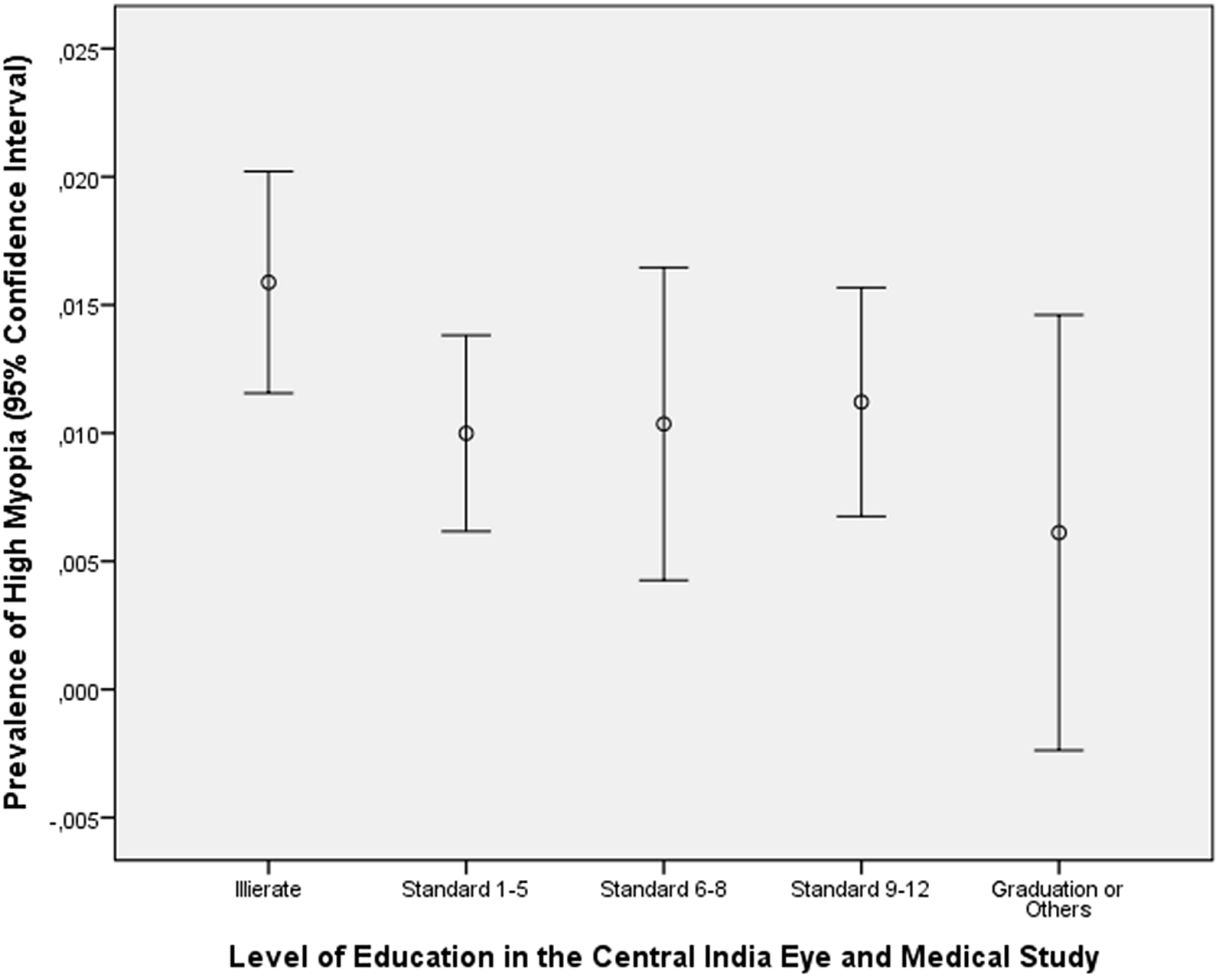
Distribution of the Prevalence of High Myopia (Defined as a Myopic Refractive Error of 6 or more Diopters), Stratified by the Level of Education in the Central India Eye and Medical Study.

In a similar manner in the Central India Eye and Medical Study, the level of education was lower, however not significantly (*P* = 0.07) lower in the highly myopic group than in the non-highly myopic group (1.12 ± 1.24 versus 1.34 ± 1.26) (Fig 2). Since the Central India Eye and Medical Study included participants with an age of 30 or more years and since a recent study by Morgan and colleagues had revealed that cycloplegia is necessary up to an age of 50 years to obtain reliable refractometric results, we performed a subgroup analysis for the Central India Eye and Medical Study by including only individuals with an age of 50+ years [24]. It revealed that again the level of education was lower, however not significantly (*P* = 0.48) lower in the highly myopic group than in the non-highly myopic group (0.81 ± 1.08 versus 1.05 ± 1.50). Correspondingly, the prevalence of high myopia was not significantly (*P* = 0.63; beta: 0.001) associated with the level of education.

**Fig 3:**
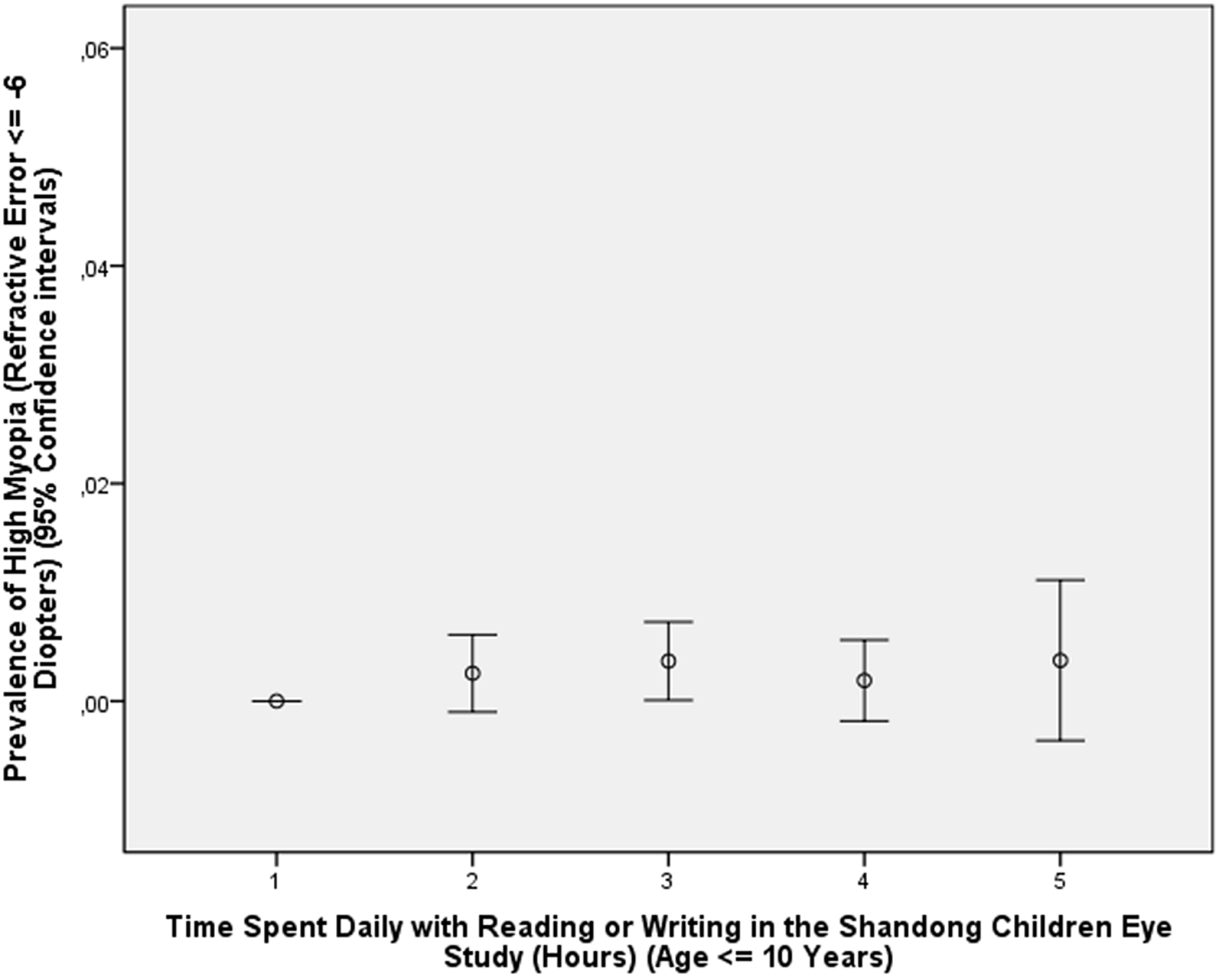
Graph Showing the Distribution of the Prevalence of High Myopia Stratified by Number of Hours Spent Indoors with Reading or Writing in the Shandong Children Eye Study in Children with an Age of ≤10 Years.

**Fig 4:**
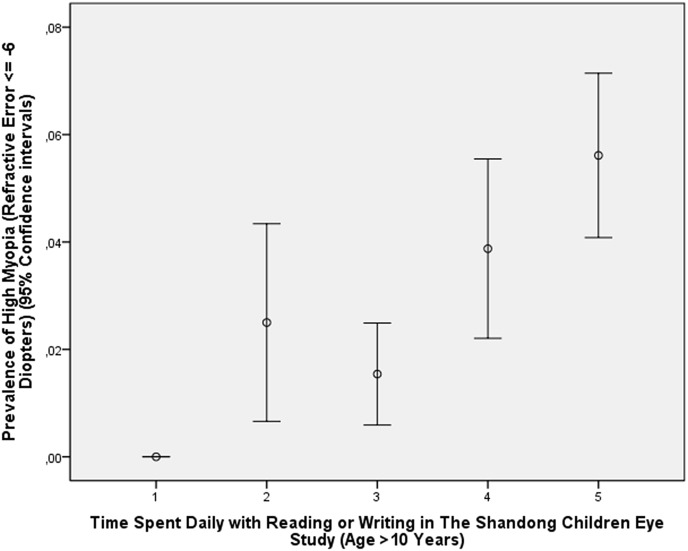
Graph Showing the Distribution of the Prevalence of High Myopia Stratified by Number of Hours Spent Indoors with Reading or Writing in the Shandong Children Eye Study in Children with an Age of >10 Years.

In the young study population of the Shandong Children Eye Study, for which cycloplegic refractometric measurements were available, a higher prevalence of high myopia was associated with higher degree of education related parameters such as higher level of maternal education (*P*<0.001) and higher level of paternal education (*P*<0.001), higher prevalence of attendance of high-level key schools versus low-level school (*P*<0.001), and more time spent for near work (*P*<0.001) (Figs 3 and 4). This effect was considerably more evident in the children aged 11+ years than in the younger group (Figs 3 and 4).

**Fig 5:**
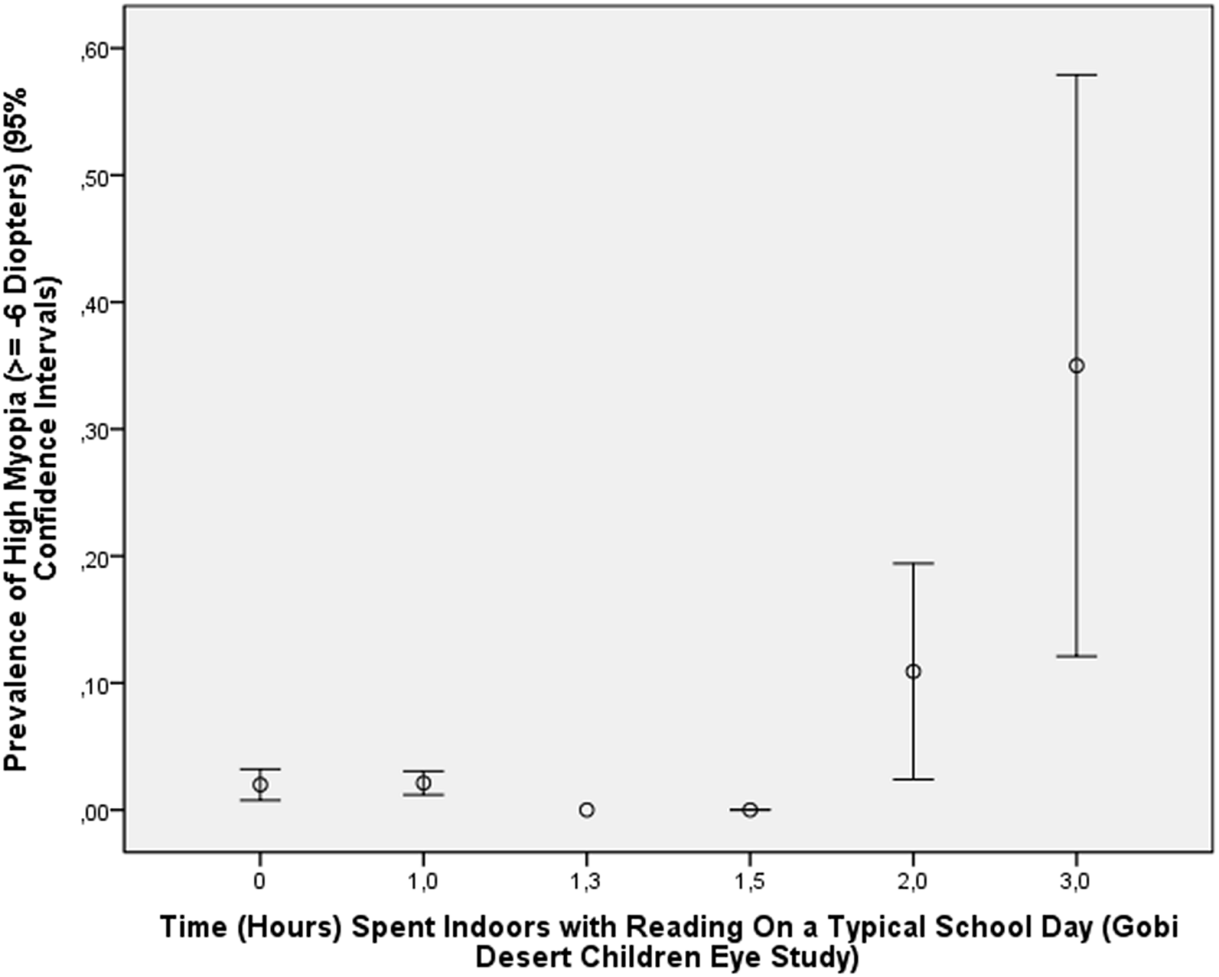
Graph Showing the Distribution of the Prevalence of High Myopia Stratified by Number of Hours Spent Daily with Reading Indoors in the Gobi Desert Children Eye Study.

In the Gobi Desert Children Eye Study, for which cycloplegic refractometric measurements were also available, a higher prevalence of high myopia was associated with more time spent indoors with reading during a typical school day (*P*<0.001) (Fig 5), more hours spent for school work (*P*<0.001), and less hours spent outdoors (*P* = 0.005).

In a similar manner in the Beijing Pediatric Eye Study, for which the AL/CCR ratio was taken as an additional surrogate for the assessment of myopia, a higher prevalence of high myopia (defined by refractometry and as defined by an AL/CCR ratio of larger than 3.25) was associated with higher degree of education related parameters such as higher level of maternal education (*P* = 0.025) and paternal education (*P* = 0.01), and more time spent for near work studying (*P*<0.001). Also in the Beijing Children Eye Study a higher prevalence of high myopia was associated with higher degree of education related parameters such as higher level of maternal education (*P*<0.001) and paternal education (*P*<0.001), higher prevalence of attendance of high-level key schools versus low-level school (*P*<0.001), and more time spent for near work (*P*<0.001). Comparable results were also obtained in the Beijing High School Teenager Study, however as in the case of the Beijing Children Eye Study, with the caveat of a potential bias by the lack of cycloplegic refractometry.

## Discussion

In the adult study populations of the Beijing Eye Study and the Central India Eye and Medical Study, the prevalence of high myopia were either not significantly associated with the level of education, or a higher prevalence of high myopia was significantly associated with a lower level of education. This was in contrast to the studies on children and teenagers, in whom a higher prevalence of high myopia was correlated with a higher degree of a variety of education-related parameters. The findings showed that the high myopia seen in older age cohorts did not reveal strong associations with the level of education, as would be expected if it were primarily genetic in origin, and the findings showed that the high myopia seen in more recent age cohorts revealed strong associations with educational parameters, as would be expected if this high myopia was a natural extension of the epidemic of myopia. It suggests two types of high myopia: The type of “acquired high myopia” which occurs in today´s school children and young adults and which is related to education-related parameters, and the type of “classic genetic high myopia” which is unrelated to close work and education associated variables and which, in contrast to acquired high myopia is more likely associated with pathological changes in the macula and optic nerve head.

The findings of our study agree with the observations made in previous investigations on children and teenager in East and South-East Asia. In these investigations, an increasing prevalence of myopia and of high myopia was associated with less time spent outdoors and more time spent indoors, higher level of schools attended, higher prevalence of parental myopia, higher level of education and higher socioeconomic standing of the parents, and urban versus rural region of habitation [8,9,25–32]. These investigations include the recent landmark studies by Wu and colleagues and by He and colleagues in which school children spending more time outdoors as compared to school children spending less time outdoors developed significantly less often myopia [29, 30]. The investigations revealed that myopia in general was associated with educational parameters, and that high myopia in recent birth cohorts was associated with educational parameters as well, whereas classical genetic high myopia was not. The observations were consistent with the notion that acquired high myopia is an extension of the overall epidemic of myopia, and that this new form of high myopia seen in more recent birth cohorts in East and Southeast Asia shows a distinctive environmental etiology and a relatively late onset. Acquired high myopia is in contrast to classical genetic high myopia, which starts at a relatively young age, which was predominant up to a time of about 20 years ago, and which appears to be less dependent on environmental parameters [7].

Confirming previous investigations, the findings of our study support the observation that the prevalence of high myopia is increasing; that the prevalence of high myopia takes off around the age of 11–13 years, as also shown in the Gobi Desert Children Eye Study and in the Shandong Children Eye Study; and that these findings suggest two forms of high myopia, a classical genetic form and an acquired form. By showing that ordinary myopia and acquired high myopia both show associations with educational parameters, the observations made in our study add to the evidence on this issue. Our study also supports the notion, what is still only speculation, that the pathological consequences of these two forms may be different, because the genetic etiology will be predominant in one form and the environmental etiology will be dominant in the other.

The results of our study should be interpreted with limitations in mind. First, it was a comparison of cross-sectional studies so that any longitudinal conclusion based on these cross-sectional data was limited in its validity. Pathological myopia usually develops at an age older than the age of the children included in our investigation. Since however myopic retinopathy usually fully develops beyond the age of 40 years, one would have had to wait 20 or more years to get longitudinal data from today´s children and teenager study populations. Second, in the group of children with high myopia, it has been difficult so far to distinguish between children with simple school high myopia (“acquired high myopia”) and children who may eventually develop pathological myopia at later age. The group of children with high myopia in the present studies therefore consisted of a mixture of both groups, so that some of the highly myopic children might have been misclassified into the group acquired high myopia. Third, a genetic analysis would have been helpful to further bolster the assumption that high myopia in today´s young generation differed from high myopia in today´s adult generation. While several studies have already revealed more than 20 genes to be associated with minor and moderate myopia, large-scaled genome-wide association studies on the genetic background of high myopia have been missing so far [33]. Fourth, categorization of the education level was different between the Beijing Eye Study and the Central India eye and Medical Study. To cite an example, the level of “half illiteracy with knowledge of some Chinese words” in the Beijing Eye Study was not same to the grade of “school visit up the 5th standard” in the Central India Eye and Medical Study, although both were graded as level “2”. It shows up differences in the questionnaires used in the various studies. Since however, the statistical analysis was performed within the various study populations and not in the total study population as a whole, differences in the study designs between the various investigations may not have markedly affected the results and conclusions of the study. Fifth, a recent study by Morgan and associates suggested that cycloplegia in population-based studies is required up to an age of about 50 years [24], so that for a substantial subgroup of the participants Central India Eye and Medical Study with an inclusion criterion of an age of 30+ years non-cylcoplegic refractometry might have shown inaccurate results. As concluded by Morgan et al., if the only important outcome measure is the prevalence of myopia, cycloplegia may not be crucial in, but without cycloplegia, measurements of other refractive categories as well as spherical equivalent could be are unreliable. In a subgroup analysis including only individuals with an age of 50+ years, however, the same results in the whole study was obtained in that the level of education was lower, however not significantly (*P* = 0.48) lower in the highly myopic group than in the non-highly myopic group. Sixth, it should be noted that all the studies on children and included into this survey reported associations between myopia and higher education parameters, but this did not invalidate the emphasis on cycloplegia, because the inaccuracy induced by the lack of cycloplegia would have been expected to make it harder to find associations. Thus, when looking at associations which were not well-documented, non-cycloplegic studies run the risk of missing associations. It is important to note that this problem is not necessarily overcome by larger sample sizes, since the errors associated with lack of cycloplegia are not linearly related to refraction (greater for hyperopic refractions) and are subject to significant inter-individual variation. Finally, the group of highly myopic children were composed of two groups, one consisting of children with genetic high myopia and one including children with acquired high myopia. The numbers suggest however, that most of the highly myopic children will be acquired high myopes since the prevalence of high myopia is low in older populations. This is also consistent with the data of Xiang and colleagues which showed only about 1% high myopia up to the age of 10, with it then rising to well over 10% [12].

In conclusion, comparing associations of old or genetic high myopia in adults with new or acquired high myopia in school children revealed that education related parameters did not show a significant association with old or genetic high myopia, while in contrast, new high myopia showed strong associations with education-related parameters. It confirms previous studies that the two forms of high myopia not only differed in age of onset, but also differed in associations with education as well. The data support the notion of two types of high myopia. Future studies may assess whether the risk of pathologic myopic maculopathy and high myopia associated open-angle glaucoma differs between both types of high myopia.

## Supporting Information

S1 Questionnaire Central India Eye and Medical Study. (DOC)

S2 Questionnaire Gobi Desert Children Eye Study. (DOC)

S3 Questionnaire Shandong Children Eye Study. (DOC)
