# Supplementary material for: Education-Related Parameters in High Myopia: Adults versus School Children
Source: PLoS One. 2016 May 6;11(5):e0154554. doi: 10.1371/journal.pone.0154554 (PMC4859491; doi:10.1371/journal.pone.0154554)
Supplement: S1 Questionnaire — (DOC) [file pone.0154554.s001.doc]

# NAGPUR EYE STUDY

# Baseline data collection- Form I

| Field Worker Name:……………………………….Date:  Village Name:…………………… Household No.: |
| --- |

I. Name of the respondent:

II. Relationship to the head of the household:

2

1

III. Type of Family: Joint Nuclear

1

2

3

IV. Religion: Hindu Muslim Christian Others, specify:………

V. Caste…

1

2

3

VI. House Ownership: Own House Rental On lease

VII. No. of Acres of land owned by the family: VIII. Settled since the past:

1

1

Less than 1 acre 6 months – 1 year

1 – 5 Acres 1 – 5 years

2

More than 5 Acres 5 – 10 years

3

2

3

4

Nil > 10 years

4

IX. Type of land:

Irrigated:

1

2

3

4

Non-Irrigated:

Both:

Not Applicable:

X. No. Of livestock:

1

Ox………………..

Cows/ Buffaloes……………….

Goat ………………

2

3

4

Other, name………………….

XI. Household article details:

| CODE | NAME OF ARTICLE | TICK |  |  |  |  |
| --- | --- | --- | --- | --- | --- | --- |
| 01 | Telephone |  |  |  |  |  |
| 02 | T.V- B.W/ Colour |  |  |  |  |  |
| 03 | Two Wheeler |  |  |  |  |  |
| 04 | Pump Set oil engine |  |  |  |  |  |
| 05 | Bullock Cart |  |  |  |  |  |
| 06 | Tractor |  |  |  |  |  |
| 07 | Refrigerator |  |  |  |  |  |
| 08 | Others, specify |  |  |  |  |  |

CODES FOR DEMOGRAPHY:

**A. Relation to head**: 01- Wife 02- Son 03 – Daughter 04- Grandchildren

05- Daughter in Law 06- Son in Law 07- Brother 08- Sister

09- Brother’s Wife 10- Father 11- Mother 12 Other…

**B**. **Sex:** 01- Male 02- Female

**C**. **Age:**

**D. Marital Status**: 01. Married 02. Unmarried

**E. Consanguinity:** 01. YES 02. NO.

**F. Education:**  01. Illiterate 02. Vth Pass 03. Passed Class VIIIth 04. Xth Pass

05. XIIth Pass 06. Graduate 07. Post Graduate

**G. Occupation:** 01-Own Agriculture 02- Agriculture coolie 03- Coolie 04- Government employee

05- Private concern 06- Business 07- Professional 08- School going

09- Not working 10- others, specify…

**H. Monthly Income:** 01. Below Poverty Line 02. Rs. 1,500/- to Rs. 5,000 03. > Rs. 5,000/-

**I. Wearing Glasses:** 01. YES 02. NO

**J. Tobacco Consumption*:** 01. YES 02. NO

**K. Alcohol Consumption:** 01. YES 02. NO

*** Tobacco includes: Beedi, Cigarettes & Pan Masala etc.**

**L.** **Ocular Disease:**  01. YES 02. NO

XII. Demographic Details of the Family Members 40 years & above.

| Sr. No. | Name of the Person | Relation to Head | Sex | Age | Martial Status | Consanguinity | Education Status | Occupation | Reported Monthly Income | Wearing Glasses | Alcohol Consumption | If **YES** Average Daily Consumption per person | How many days a week? | Tobacco Consumption* | **YES**, No. /day per person | Ocular Diseases |
| --- | --- | --- | --- | --- | --- | --- | --- | --- | --- | --- | --- | --- | --- | --- | --- | --- |
| CODES |  | **A** | **B** | **C** | **D** | **E** | **F** | **G** | H | **I** | **J** | **H** | **I** | **J** | **K** | L |
| 1. |  |  |  |  |  |  |  |  |  |  |  |  |  |  |  |  |
| 2. |  |  |  |  |  |  |  |  |  |  |  |  |  |  |  |  |
| 3. |  |  |  |  |  |  |  |  |  |  |  |  |  |  |  |  |
| 4. |  |  |  |  |  |  |  |  |  |  |  |  |  |  |  |  |
| 5. |  |  |  |  |  |  |  |  |  |  |  |  |  |  |  |  |
| 6. |  |  |  |  |  |  |  |  |  |  |  |  |  |  |  |  |
| 7. |  |  |  |  |  |  |  |  |  |  |  |  |  |  |  |  |
| 8. |  |  |  |  |  |  |  |  |  |  |  |  |  |  |  |  |
| 9. |  |  |  |  |  |  |  |  |  |  |  |  |  |  |  |  |
| 10. |  |  |  |  |  |  |  |  |  |  |  |  |  |  |  |  |
| 11. |  |  |  |  |  |  |  |  |  |  |  |  |  |  |  |  |
| 12. |  |  |  |  |  |  |  |  |  |  |  |  |  |  |  |  |
