# Supplementary material for: Education-Related Parameters in High Myopia: Adults versus School Children
Source: PLoS One. 2016 May 6;11(5):e0154554. doi: 10.1371/journal.pone.0154554 (PMC4859491; doi:10.1371/journal.pone.0154554)
Supplement: S2 Questionnaire — (DOC) [file pone.0154554.s002.doc]

### CONTACT DETAILS

Child ID

Child Name: ______________________________________________

Home phone: _______________________

Mobile phone: ­­­­­­­­­­­­­­­­­­_____________________

Email: ____________________________

### QUESTIONS ABOUT HOW YOU SPEND A TYPICAL SCHOOL/WORK DAY

1. **SCHOOL/WORK:** How many days per week do you attend school or work ⬜

*(days)*

1. **SLEEP:** When do you usually go to sleep at night? ⬜ ⬜ **.** ⬜ ⬜ *(hour) (minute)*
2. **SLEEP:** What time do you usually wake up in the morning? ⬜ ⬜ **.** ⬜ ⬜ *(hour) (minute)*
3. **BEFORE YOU LEAVE FOR SCHOOL:** After you wake up in the morning and before you leave for school/work, do you spend any time outside?

□ Not at all

□ less than an hour

More than one hour (please specify) ⬜ ⬜

*(hours)*

1. **TRAVEL:** What time do you leave home to go to school/work? ⬜ ⬜ **.** ⬜ ⬜ *(hour) (minute)*
2. **TRAVEL:** How do you travel to school/work and for how long?

□ Bus, train or tram ⬜ ⬜

□ Car ⬜ ⬜

□ Walking, bicycle or motorbike ⬜ ⬜

*(minutes)*

1. **TRAVEL:** What time do you arrive at school/work? ⬜ ⬜ **.** ⬜ ⬜ *(hour) (minute)*
2. **SCHOOL/WORK:** After you arrive at school/work, do you spend any time outside before school starts?

□ Not at all

□ less than an hour

More than one hour (please specify) ⬜ ⬜

*(hours)*

1. **SCHOOL/WORK:** What time do you usually start school/work ⬜ ⬜ **.** ⬜ ⬜ *(hour) (minute)*

1. **SCHOOL/WORK:** In the middle of your school/work day, do you spend any time outside?

□ Not at all

□ less than an hour

More than one hour (please specify) ⬜ ⬜

*(hours)*

1. **SCHOOL/WORK:** What time do you usually finish school/work ⬜ ⬜ **.** ⬜ ⬜ *(hour) (minute)*
2. **SCHOOL/WORK:** After school/work finishes, do you spend any time outside before leaving to go home?

□ Not at all

□ less than an hour

More than one hour (please specify) ⬜ ⬜

*(hours)*

1. **TRAVEL:** What time do you leave school/work to go to home? ⬜ ⬜ **.** ⬜ ⬜ *(hour) (minute)*
2. **TRAVEL:** Do you travel to home the same way as you traveled in the morning?

□ Yes

□ No, *if so how do you travel?* □ Bus, train or tram ⬜ ⬜

□ Car ⬜ ⬜

□ Walking, bicycle or motorbike ⬜ ⬜

*(minutes)*

1. **TRAVEL:** What time do you arrive at home? ⬜ ⬜ **.** ⬜ ⬜ *(hour) (minute)*
2. **AFTER YOU ARRIVE HOME:** After you arrive home and before nighttime do you spend any time outside?

□ Not at all

□ less than an hour

More than one hour (please specify) ⬜ ⬜

*(hours)*

*We would now like to ask you about how you spend your time when you are not in school or asleep. We need to know how long you are indoors or outdoors and what kinds of activities you do. We will start with indoor activities. Remember* ***do not*** *include school/work or sleep time. Indoor time can include bus, car or train travel, but not walking, riding a bicycle or motorbike.*

1. On a typical school/work day, **WHILE YOU ARE INDOORS**, for how long (per day) do you do the following activities:
2. Read printed material for pleasure, for example reading a magazine or novel?

□ Not at all

□ less than an hour

More than one hour (please specify) ⬜ ⬜

*(hours)*

1. Read printed material or do handwriting for study/work?

□ Not at all

□ less than an hour

More than one hour (please specify) ⬜ ⬜

*(hours)*

1. Use computers for study/work/pleasure?

□ Not at all

□ less than an hour

More than one hour (please specify) ⬜ ⬜

*(hours)*

1. Watch television/go to the movies?

□ Not at all

□ less than an hour

More than one hour (please specify) ⬜ ⬜

*(hours)*

1. Play sports or exercise indoors?

□ Not at all

□ less than an hour

More than one hour (please specify) ⬜ ⬜

*(hours)*

1. Are there any other indoor activities that you would do for **more than 2 hours** in a typical day?

□ Not at all

□ Yes 1. Please specify the activity ____________________ ⬜ ⬜

*(hours)*

2. Please specify the activity ___________________ ⬜ ⬜

*(hours)*

3. Please specify the activity ___________________ ⬜ ⬜

*(hours)*

1. **OUTDOORS:** How many hours do you spend outdoors in a day ⬜ ⬜ ***DO NOT*** *include school/work/sleep*  *(hours)*

1. While you are outdoors, do you do any close work activities such as; reading for pleasure or study, use computers or watch television?

□ No *(if answered no, proceed to question 18b)*

□ Yes Please specify the activity ____________________ ⬜ ⬜

*(hours)*

□ Another? Please specify the activity ________________ ⬜ ⬜

*(hours)*

1. Play sports or exercise outdoors?

□ Not at all

□ less than an hour

More than one hour (please specify) ⬜ ⬜

*(hours)*

1. Are there any other outdoor activities that you would do for **more than 2 hours** in a typical day, for example walking, gardening or shopping outside?

□ Not at all

□ Yes Please specify the activity ____________________ ⬜ ⬜

*(hours)*

□ Another? Please specify the activity ________________ ⬜ ⬜

*(hours)*

### QUESTIONS ABOUT HOW YOU SPEND A TYPICAL NON-SCHOOL/WORK DAY

1. **SCHOOL/WORK:** Do you attend any academic tuition classes, for example mathematics or language or music classes on a typical non-school/work day?

□ Not at all

□ less than an hour

More than one hour (please specify) ⬜ ⬜

*(hours)*

1. **SLEEP:** When do you usually go to sleep at night? ⬜ ⬜ **.** ⬜ ⬜ *(hour) (minute)*
2. **SLEEP:** What time do you usually wake up in the morning? ⬜ ⬜ **.** ⬜ ⬜ *(hour) (minute)*

1. **INDOORS:** How many hours do you spend indoors in a day ⬜ ⬜ ***DO NOT*** *include sleep or academic tuition classes (hours)*

1. On a typical non- school/work day, **WHILE YOU ARE INDOORS**, for how long (per day) do you do the following activities:
2. Read printed material for pleasure, for example a magazine or novel?

□ Not at all

□ less than an hour

More than one hour (please specify) ⬜ ⬜

*(hours)*

1. Read printed material or do handwriting for study/work?

□ Not at all

□ less than an hour

More than one hour (please specify) ⬜ ⬜

*(hours)*

1. Use computers for study/work/pleasure?

□ Not at all

□ less than an hour

More than one hour (please specify) ⬜ ⬜

*(hours)*

1. Watch television/go to the movies?

□ Not at all

□ less than an hour

More than one hour (please specify) ⬜ ⬜

*(hours)*

1. Play sports or exercise indoors?

□ Not at all

□ less than an hour

More than one hour (please specify) ⬜ ⬜

*(hours)*

1. Are there any other indoor activities that you would do for **more than 2 hours** in a typical day?

□ Not at all

□ Yes 1. Please specify the activity ____________________ ⬜ ⬜

*(hours)*

2. Please specify the activity ___________________ ⬜ ⬜

*(hours)*

3. Please specify the activity ___________________ ⬜ ⬜

*(hours)*

1. **OUTDOORS:** How many hours do you spend outdoors in a day ⬜ ⬜ ***DO NOT*** *include school/work/sleep*  *(hours)*

1. On a typical non-school/work day, **WHILE YOU ARE OUTDOORS**, for how long (per day) do you do the following activities:
2. While you are outdoors, do you do any close work activities such as; reading for pleasure or study, use computers or watch television?

□ No *(if answered no, proceed to question 18b)*

□ Yes Please specify the activity ____________________ ⬜ ⬜

*(hours)*

□ Another? Please specify the activity ________________ ⬜ ⬜

*(hours)*

1. Play sports or exercise outdoors?

□ Not at all

□ less than an hour

More than one hour (please specify) ⬜ ⬜

*(hours)*

1. Are there any other outdoor activities that you would do for **more than 2 hours** in a typical day, for example walking, gardening or shopping outside?

□ Not at all

□ Yes Please specify the activity ____________________ ⬜ ⬜

*(hours)*

□ Another? Please specify the activity ________________ ⬜ ⬜

*(hours)*

Comments: ________________________________________________________________

__________________________________________________________________________

__________________________________________________________________________

__________________________________________________________________________
